# Dual-task timed up and go performance and sleep quality in relation to fall history in older adults with type 2 diabetes: a cross-sectional study

**DOI:** 10.3389/fpubh.2026.1856870

**Published:** 2026-08-06

**Authors:** Xiaorui Zhang, Shan Wang, Chiyu Sun, Shushu Li, Yushi Hu

**Affiliations:** 1 School of Sports Medicine and Health, Chengdu Sport University, Chengdu, China; 2 Department of Endocrinology, Affiliated Hospital of Chengdu University, Chengdu, China; 3 Taizhou Polytechnic College, Taizhou, China

**Keywords:** type 2 diabetes mellitus, older adults, falls, dual-task, sleep quality

## Abstract

**Background:**

Emerging evidence indicates that individuals with type 2 diabetes mellitus (T2DM) have a higher incidence of falls compared to individuals without diabetes, likely due to impairments in both motor and cognitive function. To guide targeted prevention strategies, this study aims to investigate the association between TUG-DT performance, postural control, sleep quality, depression, and lifestyle habits and fall history in middle-aged and older adults with T2DM.

**Methods:**

A cross-sectional, observational study involved 60 participants aged over 50, comprising 30 patients with T2DM and 30 age- and gender-matched healthy controls. The T2DM cohort was further stratified into faller and non-faller subgroups. Assessments included: (1) mobility via TUG and TUG-DT; (2) balance and fear of falling via postural stability and the Falls Efficacy Scale International (FES-I); (3) Psychosocial and sleep status using Center for Epidemiologic Studies Depression Scale (CES-D), and Pittsburgh Sleep Quality Index (PSQI); (4) lifestyle factors, including smoking, alcohol, tea intake, and exercise status.

**Results:**

Significant differences were observed in TUG (*p* = 0.034) and TUG-DT (*p* < 0.001) time, postural stability (*p* < 0.05), visual preference (*p* = 0.045), CES-D (*p* = 0.015), and smoking (*p* = 0.01) and exercise (*p* < 0.001) behavior between T2DM and control groups. Notably, TUG-DT performance was significantly impaired in the T2DM group, with increased cognitive errors (*p* = 0.015), cognitive pauses (*p* < 0.001), and movement pauses (*p* = 0. 021). Within the T2DM group, PSQI emerged as the only variable significantly associated with fall status in logistic regression analysis [OR = 1.239, 95% CI: (1.033, 1.488)]. ROC analysis demonstrated that PSQI had acceptable discriminative ability (AUC = 0.785, *p* = 0.010), with an optimal cut-off value of 9.5, yielding a sensitivity of 72.7% and a specificity of 84.2%.

**Conclusion:**

The TUG-DT test is sensitive to detecting declines in functional mobility associated with T2DM. However, the history of falls in individuals with T2DM is primarily influenced by behavioral and psychological factors rather than isolated motor metrics. Poor sleep quality, indicated by a PSQI score greater than 9.5, emerges as a critical correlate of falls. Therefore, the risk of falls in individuals with T2DM is a multidimensional construct.

## Introduction

Falls represent the second leading cause of unintentional injury globally, with over one in four older adults experiencing at least one fall annually (1). In China, falls are the leading cause of injuries among individuals aged 65 years and older (2). Falls can result in severe consequences, including death, disability, and a decline in the ability to perform activities of daily living. These outcomes not only elevate healthcare utilization, including medical care, rehabilitation, and long-term support services, but also impose significant economic and caregiving burdens on families and society. According to the Seventh National Population Census, older adults constitute 13.5% of the total population in China, which corresponds to approximately 190.64 million individuals (3). With the rapid aging of the Chinese population, the incidence and burden of falls are expected to increase further, making fall prevention an increasingly urgent public health priority.

Over the past two decades, type 2 diabetes mellitus (T2DM) has emerged as a major global public health concern, affecting over 483 million individuals worldwide (4). China has the largest number of cases globally (5). Emerging evidence indicates that older adults with T2DM have a 1.64-fold higher risk of falls than individuals without diabetes, and are more likely to sustain fall-related injuries, particularly hip fractures (6, 7).

The elevated fall risk in older adults with T2DM is likely multifactorial. Chronic hyperglycemia can lead to peripheral neuropathy and impairments in executive function, both of which reduce attentional capacity and compromise dual-task control. In parallel, diabetes-related visual and vestibular dysfunction further compromises postural stability (8–10). Collectively, these changes reduce the capacity to allocate attention between concurrent motor and cognitive demands. This results in degraded postural control, increased gait variability, and reduced stability under complex walking conditions (11, 12). Importantly, modifiable behavioral and psychological factors—including sleep quality, depression, and physical activity—may further exacerbate this pathway by influencing attentional resources, motor control, and postural regulation (13–15). Taken together, a mechanistic cascade can be proposed: chronic hyperglycemia leads to neuropathy and executive dysfunction, which reduces attentional capacity, thereby increasing cognitive–motor interference and ultimately contributing to instability and falls.

These neurocognitive and behavioral impairments highlight the importance of assessing functional mobility under dual-task conditions. In the real world, walking rarely occurs as an isolated motor task but is typically embedded within multitask situations requiring concurrent cognitive processing, such as attention allocation and environmental monitoring (e.g., talking on the phone, engaging in social conversation, or navigating busy or unpredictable environments), thereby increasing the likelihood of dual-task interference. Traditional assessments of mobility and balance, such as the single-task Timed Up and Go (TUG) tests, may not adequately capture real-world functional demands where motor and cognitive tasks are performed simultaneously. The dual-task Timed Up and Go (TUG-DT) is a standardized laboratory-based dual-task paradigm designed to assess cognitive–motor interference. It incorporates an additional cognitive task during mobility and may provide complementary information regarding cognitive–motor interaction under more complex walking conditions (16, 17).

However, previous studies have investigated dual-task gait performance in individuals with diabetes mellitus, particularly focusing on isolated gait parameters or comparisons between individuals with and without diabetic peripheral neuropathy. In addition, limited studies have employed the TUG-DT as a primary outcome measure in older adults with T2DM (18, 19). Moreover, limited research has comprehensively examined TUG-DT performance in combination with behavioral, psychological, and postural control factors (20). Consequently, the integrated association between dual-task performance and fall-related outcomes in T2DM remains unclear. Therefore, this study aimed to (1) compare TUG-DT performance and postural control, sleep quality, depressive symptoms, and lifestyle factors between individuals with T2DM and individuals without diabetes; and (2) compare these variables between fallers and non-fallers among older adults with T2DM, and further examine the multivariable associations between these factors and fall history in this population.

## Research design and methods

### Study participants

This cross-sectional study was conducted at the Institute of Sports Medicine and Health of Chengdu Sport University in Chengdu, China. Participants were recruited between March 2022 and December 2022 from the Department of Endocrinology at the Affiliated Hospital of Chengdu University. Patients T2DM were enrolled using a consecutive sampling strategy from all eligible individuals attending the outpatient clinic during the study period. Healthy control participants were also recruited using a consecutive sampling strategy from the same hospital catchment area and community population. Controls were selected to be comparable in age and sex distribution to the T2DM group. All participants were required to be between 50 and 80 years of age.

A total of 30 patients with T2DM and 30 healthy individuals were included in the final analysis. The diagnosis of diabetes was based on the criteria outlined in the Global Guideline for T2DM (18). The exclusion criteria included: (1) type 1 diabetes or secondary diabetes; (2) contraindications to exercise; (3) severe impairments in language, vision, or hearing; (4) clinical diagnoses of severe psychiatric disorders (e.g., major depressive disorder) or dementia; (5) a history of central nervous system diseases (e.g., stroke, Parkinson’s disease) or other sensorimotor dysfunctions unrelated to diabetes; and (6) current use of medications strongly affecting central nervous system function or balance (e.g., sedatives, hypnotics, and antipsychotics) within the past 3 months.

Clinical characteristics related to diabetes were also collected for participants with T2DM. Disease duration was recorded as the number of years since diagnosis of T2DM. Information regarding current antidiabetic treatment was obtained through structured interviews and medical records, including the use of oral hypoglycemic agents and insulin therapy.

### Sample size

An *a priori* sample size calculation was performed using G*Power 3.1 based on previous studies (19) reporting significant differences in TUG-DT between older adults with prediabetes and T2DM. Using a two-tailed independent-samples t-test with *α* = 0.05 and a power (1 − *β*) of 0.90, the minimum required sample size was estimated to be 20 participants per group. Accounting for a potential dropout rate of 20%, the target sample size was increased to 25 participants per group. Ultimately, a total of 60 participants were enrolled in the study.

### Procedures

Following the collection of demographic information, the same physiotherapist (SL) conducted the outcome measurements in a consistent order. These measurements were performed in a well-lit and quiet environment, ensuring that appropriate rest intervals were provided.

Written informed consent was obtained from all participants, and the study adhered to the principles outlined in the Declaration of Helsinki.

This study was approved by the Ethics Committee of Chengdu Sport University (202119), and was registered at the Chinese Clinical Trial Registry (ChiCTR) with registration number ChiCTR2100044646.

### Measures

#### Lifestyle and orthostatic hypotension (OH) test

All participants underwent structured interviews to collect information on health status and lifestyle factors, including smoking, alcohol consumption, tea intake, exercise habits, and medication use (including insulin). Smoking status (binary; nonsmoker, current smoker), alcohol consumption (binary; nondrinker, drinker), tea intake (binary; non–tea drinker or tea drinker), and regular exercise (binary: non-regular exerciser or regular exerciser). Regular exercise was defined as engaging in physical activity for more than 30 min at least three times per week.

Given that orthostatic hypotension (OH) is a significant contributor to falls, an active bedside standing test was performed to assess OH. This condition is defined as a sustained decrease in systolic blood pressure of at least 20 mmHg or diastolic blood pressure of at least 10 mmHg within 3 min of standing (20).

#### Falls assessment

Falls were assessed using 2 questions: (1) Have you experienced a fall in the past 12 months? (2) If yes, how often did you fall during the duration of T2DM? Two dichotomous outcomes were defined: fallers (1 or more falls) and non-fallers (0 falls) in the T2DM group.

#### Timed up and go test

The test consisted of five stages: rising from a chair, walking three meters, turning around, returning, and sitting back in the chair. Each participant performed the test twice, with the time recorded using a timer. The average time taken for the test was calculated as the variable of interest (21).

#### TUG with dual-task

In the dual-task condition, participants were instructed to continuously subtract a random two-digit number between 50 and 100 by 3. The time taken to complete the task was recorded, alongside cognitive errors (the number of incorrect digits), cognitive pauses (the number of times counting was interrupted, defined as a pause lasting more than 2 s), and movement pauses (the number of walking interruptions). Each participant underwent the test twice, and the average results were calculated for subsequent analysis (22).

#### Falls efficacy scale international

Fear of falling was assessed using the Chinese version of the International Fall Efficacy Scale.

#### Postural control ability

The NeuroCom SMART Balance Master (Natus Medical Incorporated, Seattle, WA) was employed to test the Sensory Organization Test (SOT) and Limits of Stability (LOS). Participants were instructed to maintain an upright stance on the force plate, keeping their arms at sides, wearing safety harnesses.

The Sensory Organization Test (SOT) evaluates the influence of three sensory systems on postural control. Data was collected across six unique conditions, each comprising three 20-s trials. Different sensory inputs such as somatosensory (SOM), visual (VIS), vestibular (VEST), and visual preference (PREF) can be isolated. Additionally, balance maintenance abilities were assessed based on the extent of postural sway, as indicated by Equilibrium Score (ES), Balance Strategy (BS), and Composite (COMP).

(1) ES: For healthy participants, the theoretical stability threshold for maintaining balance is determined to be 12.5 degrees. The ES is calculated as follows:

$$\mathrm{ES}=\frac{12.5^{{}^{\circ}}-(\begin{array}{l} \;\text{maximum angular displacement}\\ -\text{minimum angular displacement} \end{array})}{12.5^{{}^{\circ}}}\times 100\%$$

The ES score, which ranges from 0 to 100, a higher balance score indicates greater postural stability.

(2) BS: Ranges from 0 to 100, with higher scores indicating a predominant utilization of ankle strategy.
(3) COMP: Ranges from 0 to 100, and a higher COMP indicates improved postural control capabilities.

The Limits of Stability (LOS) focused on quantifying control of the center of gravity through various sway directions. We measured Reaction Time (RT), Movement Velocity (MVL), Endpoint Excursions (EPE), Max Excursions (MXE), and Directional Control (DCL) as part of the analysis.

#### Walking tasks and gait performance

Participants underwent walking assessment in both single-task and dual-task conditions by walking across a force plate at a comfortable pace. In the dual-task condition, participants were required to walk while simultaneously counting backward in increments of three, starting from a randomly selected number between 50 and 100. The test was administered three times, and gait parameters such as speed, step length (SL), step width (SW), and step length symmetry (SYM) were recorded using the NeuroCom SMART Balance Master gait analysis system.


$$\mathrm{SYM}=\frac{|\mathrm{SL}\;\text{left}+\mathrm{SL}\;\text{right}|}{\left(\mathrm{SL}\;\text{left}+\mathrm{SL}\;\text{right}\right)}\times 100\%$$

The perfect symmetry was achieved when the SYM value was 0.

#### Cognitive function, depression, and sleep

The Montreal Cognitive Assessment Basic Scale (MoCA-BC) was utilized to evaluate the overall cognitive functioning of participants (23, 24). Depression symptoms were assessed using the 10-item Center for Epidemiologic Studies Depression Scale (CES-D) (25). The Pittsburgh Sleep Quality Index (PSQI) was employed to evaluate participants’ sleep quality (26).

### Statistical analysis

All analyses were carried out using SPSS software (Version 26.0, SPSS Inc., United States).

Descriptive statistical analysis was conducted on clinical features, with continuous variables presented as mean and standard deviation (SD) or median and interquartile range (IQR), and categorical variables presented as frequency and percentages. Data normality was assessed using the Shapiro–Wilk test. Group differences were analyzed using the Mann–Whitney U test and Student’s t-test. The chi-square (χ^2^) test was utilized to evaluate the proportion between nominal variables.

To explore factors associated with fall status among participants with T2DM, binary logistic regression analyses were performed as exploratory analysis. Candidate variables were selected based on clinical relevance and prior univariate group comparisons. Univariate logistic regression analyses were first conducted to examine the individual associations between each candidate variable and fall status. Variables showing significant associations in univariate analyses, together with clinically relevant covariates (age and MoCA-BC scores), were subsequently entered into adjusted logistic regression models. Sensitivity analyses were then performed by separately adding each potential confounder (sex, BMI, orthostatic hypotension, smoking status, exercise habits, and depressive symptoms) to the base model to evaluate the robustness of the association between PSQI and fall history. Receiver operating characteristic (ROC) curve analysis was performed for variables that remained significant after adjustment. Discriminative ability was quantified using the area under the curve (AUC), and the optimal cut-off value was determined using the Youden Index.

To examine the robustness of group differences in mobility-related outcomes, sensitivity analyses were performed by adjusting for walking speed (27). Generalized linear models (GLMs) were used for continuous gait and functional mobility variables, including step width, step length, TUG, TUG-DT, and Limits of Stability (LOS) parameters, with group status (T2DM vs. healthy controls) as the fixed factor and walking speed as a covariate. For gait symmetry (SYM), a rank-based analysis of covariance (Rank ANCOVA) was conducted as a non-parametric sensitivity analysis. SYM and corresponding walking speed values were rank-transformed before analysis, with group status entered as the fixed factor.

All statistical tests were two-sided, and a *p*-value < 0.05 was considered statistically significant.

## Results

### General characteristics

As described, all 30 T2DM patients and 30 healthy controls completed the study. No significant differences were observed in various indicators except for OH between the T2DM group and the healthy control group (*p >* 0.05; Table 1). Additionally, there was no significant difference between fallers and non-fallers in the T2DM group (*p >* 0.05; Table 1).

**Table 1:** Demographic and clinical characteristics of T2DM patients and healthy controls.

	All *N* = 60	Control group *N* = 30	T2DM group				*p* ^a^
			*N* = 30	Fallers *N* = 11	Non-fallers *N* = 19	*p* ^b^	
Age (years)	65.55 (5.41)	65.33 (3.52)	65.77 (6.86)	68.09 (4.81)	64.94 (7.55)	0.226	0.763
Female [*n*, (%)]	36 (60)	19 (60)	19 (60)	8 (72.72)	11 (63.33)	0.411	1.000
T2DM duration (y)	—	—	9.44 (7.00)	9.09 (6.17)	9.48 (7.79)	0.887	—
Insulin (YES) [*n*, (%)]	—	—	6 (20)	2 (18.18)	3 (15.79)	0.343	—
Orthostatic hypotension [*n*, (%)]	3 (5)	0 (0)	3 (10)	0 (0)	3 (18.75)	0.087	0.038*
Education (y)	12 (9–15)	12 (8.75–15)	12 (9–15)	12 (9–15)	12 (8–15)	0.474	0.782
Height (cm)	159.72 (8.51)	159.00 (7.07)	160.44 (9.81)	159.99 (11.72)	160.7 (8.87)	0.853	0.489
Weight (kg)	60.94 (9.68)	59.95 (9.15)	61.93 (10.24)	62.70 (13.84)	61.48 (7.87)	0.792	0.433
Body mass index (kg/m^2^)	23.86 (2.67)	23.69 (2.35)	24.03 (2.99)	24.09 (3.30)	23.87 (2.89)	0.720	0.627
Waist hip ratio	0.89 (0.05)	0.88 (0.05)	0.89 (0.04)	0.89 (0.04)	0.89 (0.04)	0.848	0.433
Resting blood pressure(mmHg)							
systolic blood pressure	127.96 (15.23)	129.80 (17.21)	126.13 (13.00)	128.27 (9.05)	124.89 (14.92)	0.503	0.376
diastolic blood pressure	78.9 (10.20)	79.70 (10.34)	78.1 (10.17)	77.18 (9.51)	78.63 (10.75)	0.714	0.554
Heart rate (bpm)	72.5 (8.22)	71.63 (7.87)	73.37 (8.61)	72.55 (5.28)	73.84 (10.17)	0.698	0.388

Data are presented as mean (SD), median (IQR), or number (percentage). *p < 0.05; T2DM: Type 2 diabetes mellitus; ^a^*p* value between Control Group and T2DM Group derived from student t-test or Mann–Whitney U test for continuous variables and chi-square tests for categorical variables. ^b^*p* value between fallers and non-fallers in T2DM group derived from student t-test or Mann–Whitney U test for continuous variables and chi-square tests for categorical variables.

### Fall risk and fall efficacy

Significant differences were observed in the test results for the finish time of the TUG test and TUG-DT performance (finish time, cognitive errors, cognitive pause, and movement pauses) between the T2DM group and the healthy control group (all *p* < 0.05; Table 2). Specifically, compared with healthy controls, the T2DM group showed longer TUG and TUG-DT completion times, more cognitive errors, increased cognitive and movement pauses during TUG-DT. Sensitivity analyses adjusting for walking speed showed that the between-group difference in TUG-DT completion time remained significant, whereas the difference in TUG completion time was attenuated after adjustment (Supplementary Table S1).

**Table 2:** Comparison of mobility, postural control, cognition, depression, and sleep test results of T2DM patients and healthy controls.

	T2DM group	Control group	t/U	*p* ^a^
	Mean(SD)/ Median (IQR25-75)	Mean(SD)/ Median (IQR25-75)		
TUG (s)	10.35 (8.99–11.65)	9.29 (8.57–9.96)	295.00	0.034*
TUG-DT (s)	27.62 (21.15–36.35)	14.7 (10.38–17.07)	105.00	<0.001***
Cognitive errors (N)^c^	1.00 (0,2.25)	0.25 (0,1.00)	281.50	0.015*
Cognitive pauses (N)^d^	0.25 (1.00,3.63)	0.25 (0,1.50)	199.00	<0.001***
Movement pauses (N)^e^	2.5 (1.5,3.6)	2.00 (1.00,3.00)	284.00	0.021*
FES-I	7.00 (7.00,9.25)	7.00 (7.00,7.00)	333.50	0.054
Postural stability score				
Composite	72.17 (5.50)	76.00 (5.00)	2.825	0.006**
Balance strategy	86.47 (3.69)	87.76 (3.13)	1.454	0.151
Equilibrium score	75.71 (4.07)	79.92 (4.17)	3.958	<0.001***
Sensory organization test score				
Somatosensory	97.00 (96.00–99.25)	98.00 (96.00–99.00)	−0.912	0.365
Visual	82.00 (77.00–90.25)	88.00 (77.00–90.25)	321.000	0.056
Vestibular	55.97 (12.98)	59.20 (9.72)	1.092	0.279
Visual preference	97.60 (12.20)	103.87 (11.51)	2.047	0.045*
Limits of stability				
Reaction time (s)	1.30 (0.22)	1.20 (0.26)	−1.640	0.107
Movement velocity (deg/s)	2.75 (2.10–3.6)	3.25 (1.98–4.43)	414.500	0.599
Endpoint excursions (%)	63.83 (12.81)	68.67 (14.80)	0.671	0.505
Max excursions (%)	87.00 (82.5–95.25)	87.50 (81.23–95.00)	421.000	0.668
Directional control (%)	78.00 (72.75–81.25)	77.00 (72.00–83.00)	427.500	0.739
Gait performance				
Single task				
Gait speed (m/s)	0.77 (0.20)	0.78 (0.23)	1.934	0.058
Step width (cm)	14.98 (12.52–16.95)	16.35 (14.50–16.35)	509.000	0.383
Step length (cm)	47.45 (40.00–57.28)	49.9 (42.25–64.98)	369.500	0.234
Step length symmetry	14.00 (3.00–25.25)	9.00 (2.00–18.00)	530.500	0.233
Dual task				
Gait speed (m/s)	0.29 (0.15)	0.44 (0.15)	2.370	0.021*
Step width (cm)	17.60 (14.80–19.45)	16.40 (14.45–18.55)	487.500	0.579
Step length (cm)	44.31 (15.25)	49.27 (12.62)	1.370	0.176
Step length symmetry	15.50 (6.00–36.50)	22.00 (8.00–29.50)	459.000	0.894

	Median (IQR25-75)	Median (IQR25-75)	t/U	*p*
MoCA-BC score	25.00 (21.00–27.00)	25.00 (22.00–27.00)	462.500	0.852
PSQI score	7.00 (4.00–12.25)	8.00 (4.75–12.25)	471.500	0.750
CES-D score	4.00 (1.75–10.25)	2.00 (0.00–5.25)	287.500	0.015*

	*N* (%)	*N* (%)	*X* ^2^	*p* ^b^
Current smoker	10 (33.33)	2 (6.67)	6.667	0.01**
Drinker	9 (30)	5 (16.67)	1.491	0.222
Tea drinker	22 (73.33)	16 (53.33)	2.684	0.108
Regular exerciser	5 (16.67)	12 (40)	4.022	0.045***

Data are presented as mean (SD), median (IQR), or number (percentage). *p < 0.05; **p < 0.01; ***p < 0.001; CI, Confidence interval; IQR, Interquartile Range; T2DM, Type 2 diabetes mellitus; TUG, Timed Up and Go test; TUG-DT, Cognitive dual-task TUG; FES-1, Falls Efficacy Scale International; MOCA-BC, Montreal Cognitive Assessment Basic Scale; PSQI, Pittsburgh Sleep Quality Index; CES-D, Center for Epidemiologic Studies Depression Scale. ^a^*p* value derived from student t-test or Mann–Whitney U test. ^b^*p* value derived from chi-square tests. ^c^Numbers of cognitive errors of TUG-DT. ^d^Numbers of cognitive pauses of TUG-DT. ^e^Numbers of movement pauses of TUG-DT.

### Postural control ability and gait performance

The postural stability scores were significantly lower in the T2DM group compared to the healthy control group. The average COMP score was 72.17 (95% CI: 70.11 to 74.22; *p* = 0.006) for the T2DM group and 76.00 (95% CI: 74.13 to 77.87) for the healthy control group (Table 2). A similar pattern was observed in the ES score, showing a significant difference between T2DM and healthy control (*p*<0.001; Table 2). The PREF score in the T2DM group (97.60, 95% CI: 93.05 to 102.15; *p* = 0.045) was significantly lower than that in the control group (103.87, 95% CI: 99.57 to 108.16; Table 2). However, there were no significant differences in other Sensory Organization Test (SOT) (somatosensory, visual, vestibular) and Limits of Stability (LOS) scores. Regarding gait performance, no significant differences were observed between the groups except for dual-task gait speed, which was significantly lower in the T2DM group (0.29 m/s, 95% CI: 0.23 to 0.35) compared with the healthy control group (0.44 m/s, 95% CI: 0.33 to 0.44; *p* = 0 0.021; Table 2). To account for the potential confounding effect of walking speed on gait parameters, additional adjusted analyses were performed. After adjustment for walking speed, no significant between-group differences were observed for step length, step width, or gait symmetry under either single-task or dual-task conditions (all *p* > 0.05; Supplementary Table S1).

### Cognition, sleep, depression and lifestyle

The CES-D score in the T2DM group (4.00 [95% CI 3.96 to 7.97]) was significantly higher than that in the control group [2.00 (95% CI 1.62 to 4.11); *p* = 0.015; Table 2]. However, there were no significant differences in MoCA-BC and PSQI scores between the T2DM group and the control group. The percentage of current smokers was significantly higher in the T2DM group compared to the control group (33.33% vs. 6.67%; *p* = 0.01; Table 2), and the percentage of exercisers in the T2DM group was significantly lower than that in the control group (16.67% vs. 40%, *p*<0.001; Table 2). No significant differences were observed between the T2DM group and the control group in terms of alcohol-drinking and tea-drinking status.

### Characteristics of fallers and non-fallers in T2DM

We compared the characteristics of fallers and non-fallers within the T2DM group. Independent t-tests revealed significantly higher PSQI scores in fallers than in non-fallers within the T2DM group, with a mean difference of −4.72 [95% CI (−8.23, −1.20); *t* = −2.749; see Table 3]. No other significant differences were observed between fallers and non-fallers within the T2DM group. Subsequently, a binomial logistic regression was conducted to identify factors significantly associated with fall status in T2DM, yielding *χ*^2^ = 6.605, *p* = 0.010, and explaining a moderate amount of variance (Nagelkerke’s *R*^2^ = 0.270; refer to Table 4). Furthermore, the inclusion of age and the MoCA-BC score as covariates did not lead to statistically significant changes in the *χ*^2^ values (see Supplementary Table S2). Sensitivity analyses demonstrated that the association between PSQI and fall history remained stable after adjusting for individual confounders (sex, BMI, orthostatic hypotension, smoking status, exercise habits, and depressive symptoms) with no substantial changes in effect estimates or statistical significance (see Supplementary Table S3).

**Table 3:** Comparisons between fallers and non-fallers in T2DM participants.

Test	Fall status	Mean	SD	MD	95% CI	
					Lower	Upper
TUG	Non-fallers	10.80	2.54	0.599	−1.21	2.41
	Fallers	10.20	1.89			
DT-TUG	Non-fallers	30.00	12.42	−1.776	−12.86	9.31
	Fallers	31.78	17.13			
somatosensory	Non-fallers	97.84	2.32	0.569	−1.15	2.28
	Fallers	97.27	2.00			
Visual	Non-fallers	82.37	11.13	0.186	−6.82	7.19
	Fallers	82.18	7.52			
Vestibular	Non-fallers	55.89	14.26	−0.196	−10.45	10.06
	Fallers	56.09	11.07			
visual preference	Non-fallers	97.84	14.25	0.660	−8.97	10.29
	Fallers	97.18	8.08			
Reaction time	Non-fallers	1.32	0.23	0.038	−0.13	0.21
	Fallers	1.29	0.21			
Movement velocity	Non-fallers	3.09	1.32	0.202	−0.76	1.17
	Fallers	2.89	1.10			
Endpoint excursions	Non-fallers	65.26	11.76	5.17	−4.70	15.05
	Fallers	60.09	14.30			
Max excursions	Non-fallers	85.74	10.90	3.00	−6.66	12.68
	Fallers	82.73	14.86			
Single task gait speed (m/s)	Non-fallers	0.67	0.21	−0.03	−18.52	12.41
	Fallers	0.70	0.18			
Single task Step width(cm)	Non-fallers	16.93	3.32	0.36	−1.93	2.65
	Fallers	16.57	2.12			
Single task Step length(cm)	Non-fallers	47.36	14.32	−6.09	−17.92	5.74
	Fallers	53.45	16.77			
Dual task gait speed (m/s)	Non-fallers	28.00	15.11	−0.03	−14.60	8.82
	Fallers	30.88	15.06			
Dual task step width (cm)	Non-fallers	16.23	4.37	−0.25	−3.72	3.23
	Fallers	16.48	4.68			
Dual task step length (cm)	Non-fallers	41.09	14.68	−8.79	−20.35	2.76
	Fallers	49.88	15.26			
Directional control	Non-fallers	77.31	6.38	3.95	−2.24	10.15
	Fallers	77.36	10.27			
MoCA-BC	Non-fallers	24.47	3.59	0.56	−2.07	3.20
	Fallers	23.91	3.02			
PSQI	Non-fallers	6.74	4.47	−4.72**	−8.23	−1.20
	Fallers	11.45	4.63			
CESD	Non-fallers	6.32	5.14	0.952	−3.275	5.179
	Fallers	5.36	5.95			

	Fall status	Median	IQR25-IQR75	*U*	*Z*	*p*
FES-I	Non-fallers	7.00	7.00–8.00	145.5	1.995	0.077
	Fallers	9.00	7.00–11.00			
Single task Step length symmetry	Non-fallers	14.00	8.00–30.00	*76.5*	−1.207	0.232
	Fallers	5.00	1.00–19.00			
Dual task Step length symmetry	Non-fallers	18.00	11.00–36.00	*87.5*	−0.733	0.471
	Fallers	15.00	3.00–45.00			

**p < 0.01; T2DM: Type 2 diabetes mellitus; T2DM: Type 2 diabetes mellitus; TUG: Timed Up and Go test; TUG-DT: Cognitive dual-task TUG; FES-I: Falls Efficacy Scale International; MOCA-BC: Montreal Cognitive Assessment Basic Scale; PSQI: Pittsburgh Sleep Quality Index; CES-D: Center for Epidemiologic Studies Depression Scale.

**Table 4:** Logistic regression analysis with assessments significantly associated with fall status as independent variables and fall status as the dependent variable.

	B^a^	Sig	Exp(B)^b^	95% C. I. for EXP(B)
PSQI ^c^	0.215	0.021*	1.239	1.033–1.488
Constant	−2.458	0.010	0.086	—

^a^B regression coefficient B, ^b^Exp(B) odds ratio. ^c^PSQI: Pittsburgh Sleep Quality Index; *p < 0.05.

### ROC analysis and cut-off values

**Figure 1:**
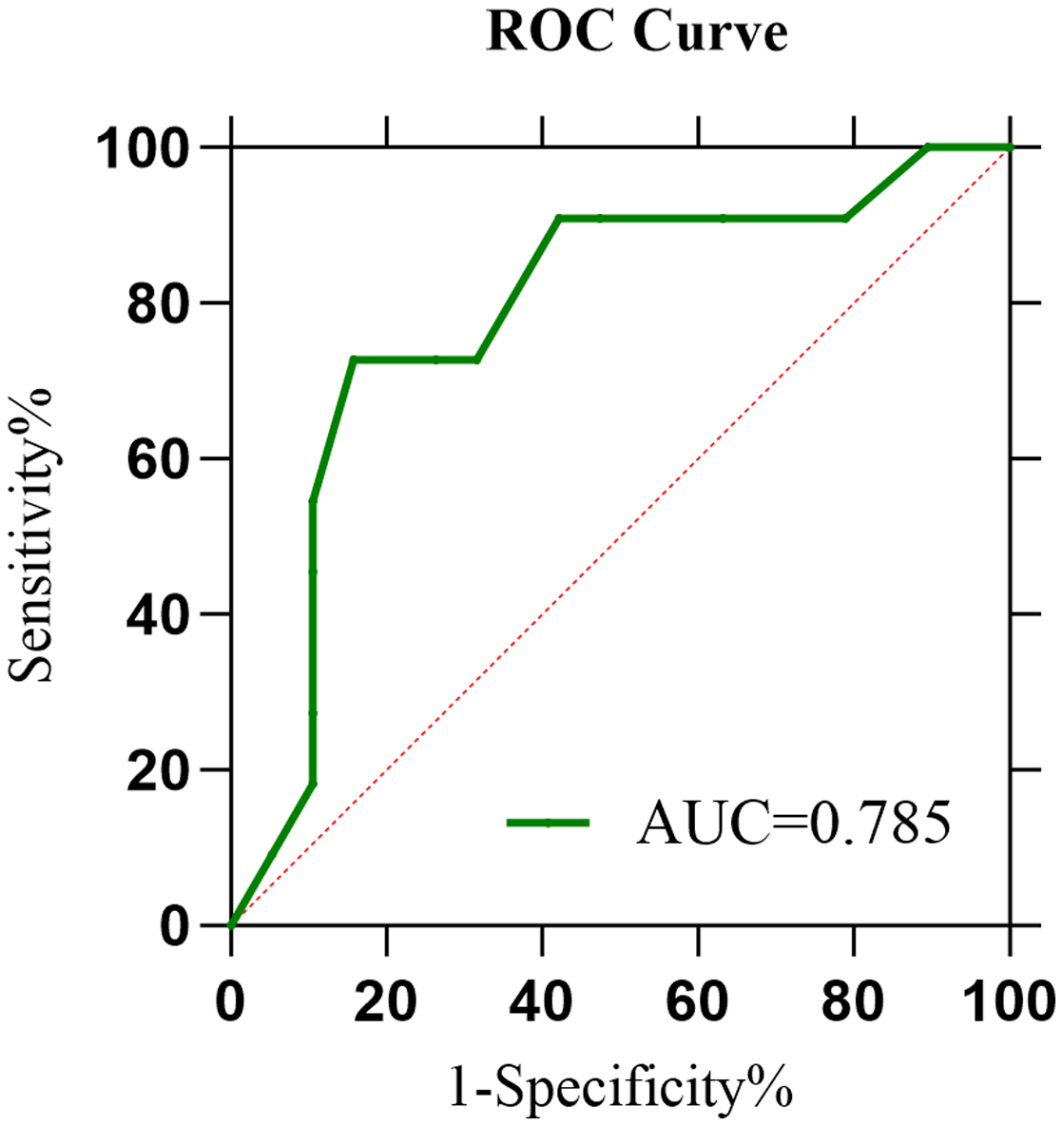
Receiver operating characteristic (ROC) curve of the Pittsburgh Sleep Quality Index (PSQI) for identifying fall history in older adults with type 2 diabetes mellitus. The area under the curve (AUC) was 0.785, and the optimal cutoff value was 9.5, with a sensitivity of 72.7% and a specificity of 84.2%.

The ROC analysis demonstrated a significant finding for the PSQI score (AUC = 0.785, *p* = 0.01). Figure 1 illustrates the ROC curves for the variables that exhibited significant AUC values. Based on the ROC coordinates and the Youden index, the optimal cut-off value for the PSQI score was identified as 9.5, yielding a sensitivity of 72.7% and a specificity of 84.2%.

## Discussion

This study provided a comprehensive evaluation of cognitive–motor performance, postural stability, sleep quality, and psychosocial risk factors in older adults with T2DM compared with healthy controls. In alignment with our primary aims, participants with T2DM demonstrated longer completion times during both the single-task TUG and TUG-DT, together with more cognitive errors and cognitive pauses during dual-task performance. Importantly, while these findings found TUG-DT may be a sensitive tool for detecting disease-related functional mobility declines, TUG-DT parameters were not significantly associated with fall history within the T2DM group. Instead, fall history was predominantly linked to prominent within-group behavioral and psychological variances, particularly sleep quality and depressive symptoms. These findings indicate that the clinical meaningfulness of our results lies not in a single, isolated predictive metric, but in recognizing that fall risk in T2DM is a multi-dimensional construct where laboratory-based mobility impairments and daily-life modifiable risks diverge.

TUG-DT captures the combined demands of motor and cognitive processing, which are commonly affected in individuals with T2DM. Importantly, sensitivity analyses adjusting for walking speed demonstrated that the between-group difference in TUG-DT completion time remained significant, suggesting that impaired dual-task mobility in individuals with T2DM cannot be fully explained by reduced walking speed alone. This finding further supports the idea that TUG-DT reflects higher-order cognitive–motor integration deficits beyond basic locomotor capacity. The observed increases in TUG-DT completion time, cognitive errors, and pauses reflect impaired attentional resource allocation. Previous studies have demonstrated that safe and independent movement, as well as the ability to perform functional tasks, heavily depend on the performance of dual-tasking abilities (28). When cognitive or sensorimotor function is compromised, greater attentional resources are required to maintain postural control, which may increase instability under dual-task conditions (29). Given that T2DM is associated with declines in executive function, processing speed, and attentional control (30), dual-task paradigms may better reflect real-world mobility demands in this population (31). However, its lack of association with fall status suggests that laboratory-based dual-task performance primarily captures maximum functional capacity under constrained testing conditions, rather than behavior under ecological conditions where falls occur.

Evidence from free-living gait studies demonstrates that real-world walking is characterized by variability, turning, and frequent transitions, with gait patterns differing substantially from those observed during straight-line walking under laboratory conditions (32–34). Wearable sensor studies further suggest that dual-task walking paradigms may better approximate real-world locomotion than single-task assessments; however, neither fully captures the complexity of daily-life gait behavior (32). This discrepancy indicates that real-world walking is not only cognitively demanding but also highly context-dependent and environmentally constrained, resulting in movement patterns that are systematically more variable and less stable than those observed in controlled laboratory settings. These findings suggest that while the dual-task Timed Up and Go (TUG-DT) provides a standardized and reproducible measure of cognitive–motor interference, it may not fully reflect the heterogeneity of mobility performance in naturalistic settings. These methodological considerations may help explain the present findings, in which TUG-DT successfully differentiated individuals with T2DM from healthy controls, yet showed limited ability to distinguish between fallers and non-fallers within the T2DM group. This pattern suggests that although TUG-DT captures cognitive–motor interference under standardized conditions, its discriminative capacity for clinically heterogeneous outcomes such as fall history may be limited.

Beyond cognitive factors, sensorimotor impairments may also contribute to the observed decline in dual-task performance. In our study, individuals with T2DM exhibited reduced postural stability and increased visual dependence compared with healthy controls, suggesting deficits in multisensory integration, which are consistent with previous research (35, 36). Postural control relies on the integration of proprioceptive, vestibular, and visual inputs. Impairments in these systems reduce the accuracy of sensory feedback, consequently increasing postural instability and sway. Whitney et al. (37) reported that individuals who experienced multiple falls had significantly lower Sensory Organization Test (SOT) composite scores. In addition, chronic hyperglycemia may impair visual function by affecting retinal microvasculature, potentially contributing to further deterioration in postural control among individuals with T2DM (38). These findings highlight that dual-task impairments in T2DM are not solely cognitive but also reflect underlying deficits in sensory integration and postural control.

Several null findings in the present study warrant balanced and clinically meaningful interpretation. Notably, no significant between-group differences were observed in global cognitive function (MoCA-BC), sleep quality (PSQI), or limits of stability (LOS) between individuals with T2DM and healthy controls. In addition, most spatial–temporal gait parameters did not differ between groups under single-task walking conditions. From a clinical perspective, the pronounced dual-task deficits observed during the TUG-DT may be more closely related to reduced efficiency in cognitive–motor resource allocation and subtle executive function limitations that are not fully captured by global cognitive screening tools such as the MoCA (39, 40). Similarly, the absence of group differences in LOS performance suggests that static or semi-static postural control assessments may have limited sensitivity to early or task-specific balance impairments in this population. Importantly, a distinct pattern emerged under dual-task conditions, where gait speed was significantly reduced in individuals with T2DM compared with healthy controls, while other gait parameters remained unchanged. This finding suggests that dual-task gait speed may be a more sensitive indicator of early cognitive–motor interference than single-task gait measures or global clinical screening tools. These results indicate that conventional single-domain assessments may not fully capture subtle functional changes associated with T2DM, whereas dual-task walking paradigms may provide greater sensitivity to early mobility-related alterations.

This study also identified behavioral and psychological factors associated with falls, in addition to functional performance. Notably, depression appears to be positively correlated with T2DM, aligning with previous evidence that suggests depression contributes to an increased incidence of falls (41). Although the direct relationship between smoking and falls remains unclear, smoking may indirectly increase fall risk through its association with reduced bone mineral density and metabolic burden (42). What’s more, physical activity plays a crucial role in fall prevention (43). Resistance training, in particular, has been shown to be more effective than aerobic exercise in glycemic control among individuals with T2DM and may represent a promising intervention strategy for fall prevention in individuals with T2DM (40, 44).

In our study, sleep quality also emerged as an important factor. A PSQI score of 9.5 emerged as a potential cutoff (AUC = 0.785) for identifying fall history in individuals with T2DM. This finding aligns with Takada’s research on community-dwelling older adults, which demonstrated that poor subjective sleep quality is longitudinally associated with falls (45). Potential mechanisms include impaired postural stability due to sleep deprivation (46), metabolic dysfunction through insulin resistance, cortisol, sympathetic activity, and inflammatory pathways (47, 48), as well as increased energy intake, body weight, and leptin/fat mass associated with reduced sleep (49). Furthermore, sleep has been shown to facilitate muscle regeneration in animal studies (50). These findings highlight the importance of incorporating sleep assessment into falls screening frameworks.

Although the FES-I scores did not differ significantly between fallers and non-fallers (*p* = 0.077), the role of fear of falling should be interpreted with caution. Previous study indicates that fear of falling independently predicts falls in individuals with T2DM under 65 years (51). While fear of falling may contribute to activity restriction and functional decline, its independent contribution to fall history requires further investigation. Overall, these findings suggest that the risk of falls in individuals with T2DM arises from the interplay of cognitive, sensorimotor, behavioral, and psychological factors, rather than being attributable to any single domain in isolation.

This study investigated TUG-DT performance combined with postural control, sleep quality, depressive symptoms, and lifestyle factors in older adults with T2DM, including a comparison with healthy controls. However, several limitations should be acknowledged. First, given the cross-sectional design, definitive causal inferences regarding the identified risk factors cannot be established, and the observed associations should be interpreted strictly as correlational. Second, the sample size for our multivariable analysis was relatively limited, as the logistic regression was inherently conducted within the T2DM subgroup. This constraint may have reduced statistical power and affected the stability of the regression estimates. Third, the comprehensiveness of our regression models was restricted by the lack of certain critical diabetes-related clinical indicators. Specifically, we did not directly evaluate diabetic peripheral neuropathy or record recent glycated hemoglobin (HbA1c) levels, preventing us from quantifying the independent impacts of sensory-motor pathways and long-term metabolic control on fall risk. It is well-established that chronically elevated blood glucose triggers advanced glycation end-products and microvascular impairments, leading to progressive somatosensory and motor nerve fiber degeneration. These neurological deficits directly compromise joint proprioception, reduce lower-limb muscular coordination, and alter mechanical gait configurations under complex walking conditions (52, 53). Finally, future prospective studies with larger clinical cohorts and longitudinal designs are strongly warranted to validate these preliminary findings, improve multivariable model robustness, and further clarify the precise mechanisms underlying fall risk in individuals with T2DM.

## Conclusion

Individuals with T2DM exhibited altered postural control and cognitive-motor performance, along with elevated levels of psychological and behavioral risk factors, including depressive symptoms, physical inactivity, and smoking behavior. The TUG-DT may serve as a valuable tool for detecting declines in functional mobility associated with T2DM. Furthermore, poor sleep quality appears to be a significant correlate of fall risk, with a PSQI score exceeding 9.5 identified as a potential threshold for recognizing individuals with T2DM who have a history of falls. Additionally, these findings suggest that falls in T2DM are multifactorial. Comprehensive clinical screening may benefit from moving beyond reliance on a single motor predictor toward a multidimensional framework incorporating dual-task mobility assessments, sleep quality evaluation, and psychological screening, thereby supporting more personalized and targeted fall-prevention strategies.

## Data Availability

The raw data supporting the conclusions of this article will be made available by the authors, without undue reservation.
