# Supplementary material for: Dual-task timed up and go performance and sleep quality in relation to fall history in older adults with type 2 diabetes: a cross-sectional study
Source: Front Public Health. 2026 Aug 6;14:1856870. doi: 10.3389/fpubh.2026.1856870 (PMC13489927; doi:10.3389/fpubh.2026.1856870)
Supplement: Supplementary file 1 [file Table_1.DOCX]

**Supplementary Table S1. Sensitivity analyses of gait parameters, Limits of Stability, and TUG performance with walking speed as a covariate**

|  | Covariate | B ^a^/ F | SE | 95% CI (Lower–Upper) | Mean Ratio Exp(B)^b^ |
| --- | --- | --- | --- | --- | --- |
| Gait Performance |  |  |  |  |  |
| Single-task |  |  |  |  |  |
| Step width | Single-task speed | 0.024 | 0.052 | (-0.078)-0.126 | 1.024 |
| Step length | Single-task speed | 0.015 | 0.051 | (-0.086)-0.115 | 1.015 |
| Step length symmetry | Single-task speed | 1.508 | — | — | — |
| Dual task |  |  |  |  |  |
| Step width | Dual-task speed | -0.021 | 0.068 | (-0.155)-0.113 | 0.979 |
| Step length | Dual-task speed | -0.106 | 0.772 | (-0.257)-0.045 | 0.899 |
| Step length symmetry | Dual-task speed | 0.825 | — | — | — |
| Limits of Stability |  |  |  |  |  |
| Reaction Time | Single-task speed | 0.066 | 0.059 | (-0.049)-0.182 | 1.069 |
| Movement Velocity | Single-task speed | -0.031 | 0.101 | (-0.228)-0.167 | 0.970 |
| Endpoint Excursions | Single-task speed | -2.753 | 3.363 | (-9.345)-3.838 | 0.064 |
| Max Excursions | Single-task speed | 0.004 | 0.033 | (-0.061)-0.068 | 1.004 |
| Directional Control | Single-task speed | 0.004 | 0.028 | (-0.051)-0.059 | 1.004 |
| TUG | Single-task speed | 0.037 | 0.042 | (-0.045)-0.120 | 1.038 |
| TUG-DT | Single-task speed | 0.533 | 0.107 | 0.322-0.743*** | 1.703 |

*Note.* Additional analyses were performed to account for the potential influence of walking speed on gait parameters. For step width (SW) and step length (SL), generalized linear models were fitted with group status (diabetes vs. non-diabetes) as the fixed factor and the corresponding walking speed as a covariate; For gait symmetry (SYM), rank-based analysis of covariance (Rank ANCOVA) was performed using ranked SYM values and ranked walking speed as the covariate; For TUG, TUG-DT, and Limits of Stability (LOS) variables, generalized linear models (GLMs) were fitted with group status as the fixed factor and single-task walking speed as a covariate.

^a^ B regression coefficient B.

^b^ Exp(B) the ratio of expected means associated with group status after adjustment for walking speed.

*** *P* < 0.001.
