# Supplementary material for: Dual-task timed up and go performance and sleep quality in relation to fall history in older adults with type 2 diabetes: a cross-sectional study
Source: Front Public Health. 2026 Aug 6;14:1856870. doi: 10.3389/fpubh.2026.1856870 (PMC13489927; doi:10.3389/fpubh.2026.1856870)
Supplement: Supplementary file 2 [file Table_2.DOCX]

**Supplementary table S2 Regression model with age and MoCA score as covariates**

|  | B^a^ | Sig | Exp(B)^b^ | 95% CI for EXP(B) | |
| --- | --- | --- | --- | --- | --- |
|  |  |  |  | Lower | Upper |
| PSQI ^c^ | 0.224 | 0.020* | 1.251 | 1.036 | 1.510 |
| Age | 0.107 | 0.217 | 1.113 | 0.939 | 1.319 |
| MoCA-BC ^d^ | -0.081 | 0.542 | 0.922 | 0.712 | 1.196 |
| Constant | -7.732 | 0.211 | 0.000 |  |  |

*Note.* This model is statistically significant (χ²(3) = 8.700, p = 0.034, Nagelkerke’s *R*² =0.252);

^a^ B: Regression coefficient B, ^b^ Exp(B): Odds Ratio ^c^ PSQI: Pittsburgh Sleep Quality Index, ^d^ MoCA-BC: The Montreal Cognitive Assessment Basic Scale

**P* < 0.05;
