# Supplementary material for: Dual-task timed up and go performance and sleep quality in relation to fall history in older adults with type 2 diabetes: a cross-sectional study
Source: Front Public Health. 2026 Aug 6;14:1856870. doi: 10.3389/fpubh.2026.1856870 (PMC13489927; doi:10.3389/fpubh.2026.1856870)
Supplement: Supplementary file 3 [file Table_3.DOCX]

**Supplementary table S3**. **Sensitivity analyses assessing the robustness of the association between PSQI and fall history in**

**T2DM**

| Model | Adjusted variables | Exp(B) ^a^ (PSQI)^b^ | 95% CI for Exp (B) | P |
| --- | --- | --- | --- | --- |
| Model 1 | Age + MoCA ^c^ | 1.251 | 1.036–1.510 | 0.020* |
| Model 2 | Model 1 + Sex | 1.248 | 1.025–1.520 | 0.027* |
| Model 3 | Model 1+ BMI ^d^ | 1.256 | 1.035–1.525 | 0.021* |
| Model 4 | Model 1 + OH ^e^ | 1.274 | 1.041–1.560 | 0.019* |
| Model 5 | Model 1 + smoking | 1.256 | 1.036–1.522 | 0.020* |
| Model 6 | Model 1 + exercise | 1.221 | 1.005–1.482 | 0.045* |
| Model 7 | Model 1 + depression | 1.271 | 1.042–1.551 | 0.018* |

Note: Due to the limited sample size and number of outcome events, a fully adjusted multivariable logistic regression model including all potential confounders was not feasible. Therefore, sensitivity analyses were performed by sequentially adding individual covariates to the base model (age + MoCA) to assess the robustness of the association between PSQI and fall history.

^a^ B: Regression coefficient B; ^b^ PSQI: Pittsburgh Sleep Quality Index; ^c^ MoCA: Montreal Cognitive Assessment; ^d^ BMI: body mass index; ^e^ OH: orthostatic hypotension.

**P* < 0.05
